# Photoinactivation vs repair of photosystem II as target of thermal stress in epipelic and epipsammic microphytobenthos communities

**DOI:** 10.1371/journal.pone.0292211

**Published:** 2023-09-28

**Authors:** Cláudia Bártolo, Silja Frankenbach, João Serôdio

**Affiliations:** CESAM – Centre for Environmental and Marine Studies and Department of Biology, University of Aveiro, Aveiro, Portugal; University of Hyderabad School of Life Sciences, INDIA

## Abstract

Microphytobenthos (MPB) inhabiting intertidal flats are exposed to large and sudden changes in temperature, often simultaneously with exposure to direct sunlight. These conditions are expected to negatively impact photosynthesis by exacerbating the photoinhibition under high light. This study addressed the photoinhibitory effects of short-term exposure to cold (5°C) and moderate heat (35°C) on MPB dominated by motile epipelic (EPL) and immotile epipsammic (EPM) diatom species, by evaluating the seasonal variation of photoinactivation and repair of photosystem II (PSII). The susceptibility to PSII photoinactivation and the counteracting repair capacity were measured by the constant rates k_PI_ and k_REC_, respectively. The photoacclimation state was characterized by hysteresis light-response curves (HLC) of the relative electron transport rate, rETR, and of the nonphotochemical quenching index Y(NPQ). Under non-stress conditions (20°C), k_REC_ was on average almost 10x higher than the corresponding k_PI_ (20.4 vs 2.70 × 10^−4^ s^−1^, respectively), indicating the operation of efficient repair mechanisms. Overall, the exposure to low and high temperatures affected both PSII photoinactivation and repair but causing smaller impacts in the former than in the latter. Also, cold stress caused larger effects on repair (decrease of k_REC_) than on photoinactivation (increase of k_PI_), but heat stress affected similarly the two processes. These effects varied seasonally, suggesting a role of thermal acclimation, as heat stress had stronger effects in cold-acclimated samples and cold stress resulted in stronger effects in heat-acclimated samples. The changes in k_PI_ and k_REC_ occurred despite the high light-acclimated phenotype found all year round, indicating that these processes vary independently from the photoacclimation state. The results also showed that photoprotection processes, as measured by energy-dependent non-photochemical index q_E_, appear to have an important role, both by preventing PSII photoinactivation and by alleviating the impacts on PSII repair under acute thermal stress.

## Introduction

Much of the interest in the study of microphytobenthos (MPB) stems from its role as main contributor to the primary productivity of estuarine and shallow subtidal coastal areas. MPB communities are particular abundant in intertidal sediment flats, where they support high rates of photosynthetic carbon fixation [1, 2], as well as several associated ecosystem services [3]. The interest in MPB has been further fueled by the apparent contrast between the unique combination of extreme conditions that shape their sedimentary habitat and commonly observed high photosynthetic activity and productivity. In fact, most photosynthesis of intertidal MPB occurs during diurnal low tide, during which the microalgae are subjected to large and rapid fluctuations in the main abiotic factors [4–6], including the exposure to supersaturating irradiances for prolonged periods [7, 8], extreme high and low temperature [9, 10], extreme salinities [8] and desiccation [11]. Additionally, the sedimentary environment is characterized by steep vertical physico-chemical gradients and a very thin photic zone [1] which, in combination with frequent bioturbation and resuspension/deposition, cause the displacement of large amounts of microalgae to the aphotic layers of the sediment [12].

One way these conditions may cause significant limitations on photosynthesis and growth is through photoinhibitory damage of the photosynthetic apparatus during high light periods, exacerbated by extreme levels of other abiotic factors like temperature or salinity. Photoinhibition causes the net loss of active photosystem II (PSII; see Table 1 for notation) units, leading to a light-induced loss of photosynthetic performance and associated capacity for photochemical generation of ATP and NADPH [13]. The detrimental effects of photoinhibition of photosynthesis and productivity depend on the balance between the photoinactivation of PSII, associated to the degradation of the protein D1, and the continuous counteracting repair of damaged PSII units, through the *de novo* synthesis of this protein [14]. The PSII repair involves the energetically expensive removal and replacement of damaged protein subunits, adding to the high metabolic costs of the photoinhibitory process [13, 15].

**Table 1:** Notation.

Parameter	Description
α	Initial slope of a rETR vs. E curve (μmol quanta^-1^ m^2^ s)
ΔF/F_m_’	Effective quantum yield of PSII
E	PAR irradiance (μmol quanta m^-2^ s^-1^)
E_50_	E level for 50% of Y(NPQ)_m_ in a Y(NPQ) vs. E curve (μmol quanta m^-2^ s^-1^)
E_k_	Light-saturation parameter of a rETR vs. E curve (μmol quanta m^-2^ s^-1^)
F	Steady-state fluorescence emitted under ambient light
F_m_, F_m_’	Maximum fluorescence measured in dark- and light-acclimated samples
F_v_/F_m_	Maximum quantum yield of PSII
%F_v_/F_m_	Relative variation of F_v_/F_m_ following light exposure
H	Hysteresis index
HLC	Hysteresis light-response curve
k_PI_, k_REC_	Rate constants of photoinactivation and repair of PSII (s^-1^)
n	Sigmoidicity coefficient of the Y(NPQ) vs. E curve
NPQ	Non-photochemical quenching
Φ_PI_	Relative quantum yield of photoinactivation (m^2^ μmol quanta^-1^)
PAR	Photosynthetically Active Radiation
PSII	Photosystem II
q_E_	Energy-dependent quenching
rETR	Relative electron transport rate of PSII
rETRm	Maximum rETR in a rETR vs. E curve
XC	Xanthophyll cycle
Y(NPQ)	Regulated thermal energy dissipation related to NPQ
Y(NPQ)_m_	Maximum Y(NPQ) value of a Y(NPQ) vs. E curve
Y(NPQ)_0_	Y(NPQ) value for E = 0 during a light-decreasing Y(NPQ) vs. E curve

The photoinactivation and repair of PSII have different ecophysiological implications, being often of interest to study and quantify these two processes separately. This is typically achieved through the measurement of the rate constants of PSII photoinactivation (k_PI_) and of PSII repair (k_REC_), usually by quantifying the decrease over time of photosynthetic oxygen evolution or of the maximum quantum yield of PSII (the chlorophyll fluorescence index F_v_/F_m_) on untreated samples and on samples treated with inhibitors of protein synthesis (e.g. lincomycin) [16]. Physiologically relevant photoinhibition of PSII occurs when the rate of PSII photoinactivation exceeds the rate of PSII repair. However, the net impact of photoinhibition on photosynthetic performance will depend on the efficiency of various photoprotective processes that contribute to maintain k_PI_ lower than k_REC_. These processes can be classified in two main groups, depending on their nature and form of action: processes that limit the amount of light energy that is absorbed by the photosynthetic apparatus, and processes that limit the photodamage caused by absorbed energy [17].

The high rates of photosynthesis achieved by the MPB under the extreme conditions of the estuarine intertidal environment has been attributed to the combined operation processes of the two types. On one hand, the behavioral regulation of light exposure by motile diatoms, the group of microalgae that dominates MPB communities: through vertical migration across the thin photic zone of the sediment, diatom cells are able to select light conditions that optimize photosynthesis while minimizing photodamage, an idea encapsulated in the ‘behavioral photoprotection’ or ‘behavioral photoregulation’ hypothesis [18–20]. On the other hand, the regulated dissipation of absorbed light energy, mainly through the non-photochemical quenching (NPQ) of chlorophyll fluorescence (energy-dependent quenching, q_E_) mediated by the xanthophyll cycle [21, 22]. It was long hypothesized that the two types of processes might not operate independently from each other, but that an interplay between motility-based and physiological photoprotective mechanisms could occur, in the form of a trade-off between the two: having the ability to regulate the experienced light through vertical migration, motile diatoms possess a reduced physiological photoprotective capacity in comparison with immotile forms [22–24]. These hypotheses prompted a number of studies on the relative importance of behavioral and physiological protection in MPB [22, 23, 25–29]. An approach commonly followed to evaluate the role of diatom motility has been to compare epipelic (EPL) and epipsammic (EPM) diatoms, species that are closely related taxonomically but with distinct capacity for directed motility: the former are biraphid pennates, motile and dominant in fine sediments, while the latter are predominantly non-motile or slowly motile forms that inhabit coarser sediments [22, 30–35]. Recent studies support the motility-physiology trade-off. In comparison with EPM forms, EPL forms (i) show a weaker physiological photoprotection capacity, evaluated both in terms of cellular pools of xanthophyll cycle (XC) energy-dissipating pigments [25, 30, 32] and of maximum NPQ [22, 28, 36], and (ii) are more susceptible to photoinactivation and less dependent on the XC for preventing photodamage, relying more on vertical migration and PSII repair [31].

Another process that may affect the susceptibility to PSII photoinactivation and repair capacity is photoacclimation [37–39]. Photoacclimation allows for the regulated adjustment of phenotypical functional traits (e.g., pigment content, thylakoid stacking) to improve the match between photosynthetic performance and the experienced growth light environment. Changes in the photoacclimation state can alter the susceptibility to PSII photoinhibition, through changes in cellular pigment content, including chlorophyll *a* and photoprotective xanthophyll cycle pigments, and in the efficiency of excitation delivering to the PSII reaction center [13, 38], and through changes in the efficiency of PSII repair, by changes in the rate of D1 protein synthesis [14]. In comparison to acclimation to high light conditions, acclimation to low light tends to increase PSII photoinactivation and to decrease PSII repair capacity [40–42], often leading to photoinactivation rates that exceed those of counteracting repair [40, 43–45]. However, it has also been reported that an increase in growth light may lead to a decrease in k_PI_ [46, 47].

In the intertidal flats inhibited by MPB, exposure to high light is often accompanied by the exposure to large and sudden changes in temperature, which may reach extreme low and high levels [5, 8, 10]. This is because light exposure is mostly restricted to low tide periods, when the air-exposed sediment undergoes heat exchanges with the atmosphere, typically suffering after tidal ebb a rapid cooling during winter days and a rapid warming during summer days [4]. Temperature has been recognized as a main abiotic controlling factor of the photosynthesis of intertidal MPB and the exposure to extreme low and high temperature conditions is expected to exacerbate the photoinhibitory effects of high light and target differently the PSII photoinactivation and repair processes. Furthermore, it is expectable that variations in ambient temperature cause changes in the thermal acclimation state of microphytobenthic cells, altering their responses to high light under cold or heat conditions. Despite the expected importance of extreme temperatures on MPB photoinhibition, available studies have not distinguished PSII photoinactivation and repair [8, 48] or, when quantifying them separately, did not address the effects of temperature [31]. Also, both the effects of photo- and of thermal acclimation on k_PI_ or k_REC_ were never studied on natural MPB communities.

This study addressed the effects of cold and moderate heat on the PSII photoinactivation and repair of natural MPB. The effects were assessed by quantifying the respective rates k_PI_ and k_REC_ on communities with different motility capacity (EPL and EPM), collected at four different occasions along one year to cover for possible naturally occurring changes in photo- and thermal acclimation state. The main objectives were: (i) quantify the effects of thermal stress (cold and moderate heat) on PSII photoinactivation and on repair in natural EPL and EPM communities, to better understand if cellular motility may affect the balance between the two processes under abiotic stress; (ii) evaluate the possible effects of photo- and thermal acclimation state on the photoinhibitory responses under cold and moderate heat; (iii) characterize the dependency between PSII photoinactivation and repair, and photoprotective capacity, and how it varies under extreme temperatures.

## Materials and methods

### Sampling

Sediment samples were collected in two sedimentary intertidal sites of the Ria de Aveiro (northwest coast of Portugal) known to harbor MPB communities dominated by pennate diatoms, one characterized by fine sediment particles and dominance of EPL diatom species (Vista Alegre, 40° 35’ 00’’ N, 08° 41’ 15’’ W; hereafter VA-EPL) and the other comprised of sandy mud and dominated by EPM diatom species (Gafanha da Encarnação, 40° 37’ 34’’ N, 08° 44’ 14’’ W; hereafter GE-EPM) [20, 31]. Details about the sampling sites (location, tidal height, sediment characteristics) can be found in [12]. Sampling was carried out on four occasions from mid-Autumn of 2020 to mid-Summer of 2021, to cover one full seasonal cycle. The dates of sampling were the following: 17–18 November, 1–2 February, 28–29 April, 21–22 July. For each sampling period, the site VA-EPL was sampled on the first day and GE-EPM on the following day. Sampling of different days was necessary as it was not possible to carry out the laboratory measurements for the two sampling sites on the same day. Sampling was always done during diurnal low tide of spring tides, which for these sites corresponds to the middle of the day. No permits were required to access the field site where samples were collected.

All measurements were carried out on cell suspensions, prepared from sediment samples as described by [31]. Sediment was sieved by a 1 mm mesh and kept immersed overnight in filtered natural seawater collected at the sampling site. All photophysiological measurements were carried out on the next day. For taxonomical identification, cells were collected using the ‘lens tissue’ technique of [49], by applying two layers of lens tissue (lens cleaning tissue 105, Whatman) on the surface of the sediment while exposed to low white light (150 W halogen lamp, 80 ± 10 μmol quanta m^-2^ s^-1^) for 2 h. The cells were collected from the upper piece of lens tissue, resuspended in filtered natural seawater.

### PSII photoinactivation and repair

The susceptibility to PSII photoinactivation and the counteracting repair capacity were evaluated by measuring the constant rates of PSII inactivation and repair k_PI_ and k_REC_, respectively. The two rate constants were determined by running light stress-recovery experiments (LSE) and measuring the relative decrease in the maximum quantum yield of PSII caused by exposure to high light, %F_v_/F_m_ (ratio between post- and pre-stress F_v_/F_m_ values; see below), in untreated (control) and lincomycin-treated replicated cell suspensions, as described by [16]. Under the assumptions that (i) the reduction of the pool of functional PSII can be estimated by the light-induced change of F_v_/F_m_ and (ii) PSII photoinactivation and repair occur simultaneously and are described as two opposite first-order reactions [50, 51], %F_v_/F_m_ can be related to k_PI_ and k_REC_ through:

$$\%F_{v}/F_{m}=\frac{k_{REC}+k_{PI}e^{-(k_{REC}+k_{PI})T}}{k_{REC}+k_{PI}}$$ (1)

where T is the duration of high light exposure during the LSE. Using lincomycin as an inhibitor of the *de novo* synthesis of the chloroplast-encoded PSII protein D1 and repair of photoinactivated PSII, k_PI_ was determined from Eq (1) and %F_v_/F_m_ measured on lincomycin-treated samples, considering k_REC_ = 0. Using the value of k_PI_ thus calculated (average of three replicates, see below), k_REC_ was estimated by solving Eq (1) numerically using MS Excel Solver.

LSE consisted of: (i) a pre-stress period (15 min, dark), (ii) a light stress period (45 min, 1020 μmol quanta m^-2^ s^-1^), and (iii) post-stress period (15 min, dark). During the pre- and post-stress periods, F_v_/F_m_ was measured every 5 min. k_PI_ and k_REC_ were calculated using Eq (1) on the basis of the last F_v_/F_m_ values measured during the pre- and post-stress periods. Lincomycin (lincomycin hydrochloride; Alfa Aesar, Germany) was added in the dark 30 min before the start of the LSE. A stock solution was prepared, and its pH was adjusted to the pH found at the sampling site using NaOH (0.14 M, Sigma-Aldrich). The final lincomycin concentration was 2 mM.

### Photoprotection capacity

The values of post-stress F_v_/F_m_ measured after the 15 min relaxation on untreated samples, expressed as a percentage of pre-stress levels, were used to estimate the energy-dependent non-photochemical index, q_E_, and to evaluate the photoprotective capacity [48].

### LSE and effects of temperature

**Fig 1:**
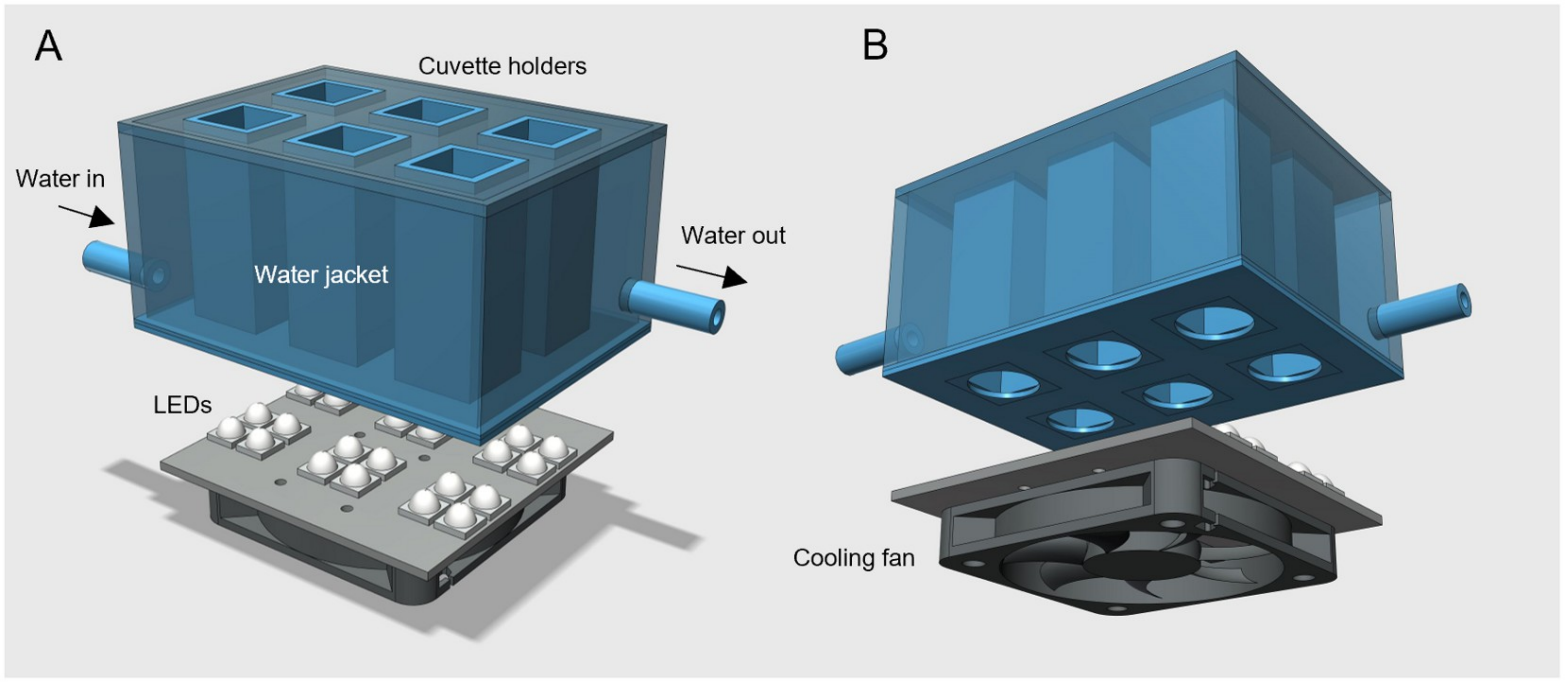
Illumination system. Exploded view of the illumination system used in this study, showing its main components. (A) Custom-designed, 3D-printed multi-cuvette holder and water jacket (blue), and square 8 × 8 SMD LED panel with individual 30° lenses (grey; only the 6 groups of 4 LEDs used for illuminating the cuvettes are shown). The water jacket was connected to a water bath allowing maintaining the 6 cuvettes at the desired temperature while being simultaneously illuminated from below. (B) Fan to dissipate the heat generated by the LEDs, placed beneath the LED panel. For simplicity, the holders used to fix the LED panel to the cuvette holder and the fan to the LED panel were omitted. The STL file for the 3D-printed water jacket is available as (S1 File). Details on the LED emission spectra and control, as well as other 3D-printed parts, are provided in [52].

A custom-made setup was used to run multiple LSE in parallel under controlled temperature conditions (Fig 1). It comprised a 3D-printed holder for six 10 x 10 spectrophotometer cuvettes mounted on a water jacket connected to a thermostatic water bath (P selecta, Frigiterm, Spain), and a RGBW LED panel illuminating the samples from below. The STL file for the 3D-printed cuvette holder and water jacket is available as (S1 File). To increase the light intensity delivered to the samples, each cuvette was illuminated by four LEDs. The LEDs were controlled by an Arduino Uno R3 microcontroller (http://www.arduino.cc), providing a mixture of white, red, green and blue light, corresponding to a PAR of 1020 μmol quanta m^-2^ s^-1^, as measured with a calibrated submersible spherical US-SQS/L micro-quantum sensor (Heinz Walz GmbH, Germany), positioned at mid-height of a cuvette filled with distilled water (1.25 ml, same volume as the cell suspensions). Details about the spectra and the controlling of the LEDs are given by [52]. The six cuvettes were stirred during the entire LSE using 5×2 mm magnetic bars and by placing the cuvette holder on top of a magnetic stirrer (Stuart CB162, Bibby Sterilin Ltd, UK) positioned below. For measuring F_v_/F_m_, each cuvette was briefly removed from the holder and placed in the optical unit of a chlorophyll fluorometer (see below), and immediately returned. This setup allowed to run six LSEs simultaneously (three untreated and three lincomycin-treated samples) and to determine k_PI_, k_REC_ and q_E_ for cell suspensions exposed to 5, 20 and 35°C on the same day (for each sampling site, day after collection). Samples were first exposed to each temperature for 15 min before the start of the corresponding LSE.

### Photoacclimation state

The photoacclimation state of the cell suspensions was characterized by measuring hysteresis light-response curves (HLC; [53]) of the relative electron transport rate, rETR, and of the nonphotochemical quenching index Y(NPQ), given respectively by:

$$rETR=E\;\frac{F_{m}^{'}-F}{F_{m}^{'}}$$ (2)

and

$$Y\left(NPQ\right)=\frac{F}{F_{m}^{'}}-\frac{F}{F_{m}}$$ (3)

where E is the incident irradiance, F_m_ and F_m_ʹ are the maximum fluorescence measured of dark-adapted and illuminated samples, respectively, and F is the steady-state fluorescence emitted under ambient light. HLCs were generated by sequentially applying eight levels of actinic light (6, 19, 74, 167, 473, 791, 1390, 2196 μmol quanta m^-2^ s^-1^) first in increasing order (light-increasing phase) and then in decreasing order (light-decreasing phase). Samples were dark acclimated for 15 min before the start of the measurements and were then exposed to each light level for 120 s throughout the entire HLC. HLCs of rETR and of Y(NPQ) were characterized by fitting the model of [54] and of [55], respectively, using a procedure written in Microsoft Visual Basic and based on Microsoft Excel Solver [31]. On some occasions, it was observed ‘dark NPQ’, i.e., values of Y(NPQ) > 0 for low light levels. In these cases, for model fitting purposes, the values of Y(NPQ) for E levels lower than the one corresponding to Y(NPQ) = 0 were considered as null, forcing the model to only characterize the high light-induced NPQ processes. The models were fitted separately to the light-increasing and light-decreasing parts of each HLC. rETR HLCs were characterized by estimating the initial slope, α, the maximum rETR level, rETR_m_, and the photoacclimation parameter, E_k_. Y(NPQ) HLCs were characterized by estimating the maximum Y(NPQ) level, Y(NPQ)_m_, the irradiance level for 50% Y(NPQ)_m_, E_50_, and the sigmoidicity coefficient, n. When the values of Y(NPQ) did not reach zero during the light-decreasing phase of the HLC, curves the model of [55] was modified as described in [53]. The magnitude and direction of the hysteresis were quantified by the non-parametric hysteresis index H, based on the normalized difference between the upward and downward phases of each HLC [53]. Three replicated HLCs were measured for each sampling site on each sampling occasion, each during the period of high light exposure of the three LSE carried out to measure k_PI_, k_REC_ and q_E_ under each tested temperature (section above).

### Chlorophyll fluorescence

All chlorophyll fluorescence measurements were carried out using a Multi-Color PAM fluorometer (Heinz Walz GmbH) with an MCP-D detector unit fitted with an RG 665 long pass filter (>650 nm, 3 mm RG665, Schott), and controlled by the PamWin V3.12w software. Blue light peaking at 440 nm was used for the measuring light and white light was used for actinic light and the saturating light pulses (800 ms). The fluorescence was measured in in 10×10×45 mm acrylic cuvettes (Sarstedt, Germany) using the ED-101US/MD optical unit, coupled to a magnetic stirrer (PHYTO-MS Miniature Magnetic Stirrer, Walz). The fluorometer was zeroed using filtered seawater as a blank.

### Taxonomical composition

Sub-samples of the cell suspensions were fixed in Lugol’s solution (5% v/v) (5% iodine, AppliChem; ITW Reagents; USA) and viewed under a bright-field microscope for determination of the relative abundance of major taxonomic groups (diatoms, euglenophytes and cyanobacteria). A Naegeotte cell counting chamber (Blau Brand; Germany) was used to count a minimum of 300 cells at 40x magnification. Diatom identification was performed on sub-samples oxidized using concentrated nitric acid (1/4 v/v) and potassium permanganate. Fully oxidized samples were then mounted on a microscopy slide using Naphrax (Northern Biological Supplies, UK) to prepare permanent microscopy slides. Taxa identification was performed at 100x magnification with a minimum number of individual valves of at least 700 cells or valves for each site and sampling occasion. Diatom identification was based on morphological features of the valves, including its shape, length and width but also raphe presence, length and shape, patterns within the striae, and ornamentation features of the frustule [56–58].

### Statistical analyses

Measurements made on different sampling dates and sites were compared by applying two- or three-way Analysis of Variance (ANOVA), and by post hoc Tukey HSD test. Assumptions of normality and homoscedasticity were verified prior to analysis using the Shapiro–Wilk test and Levene’s test, respectively. In case of violation of assumptions, data were log transformed. All statistical analyses were carried out using SPSS Statistics 142 (IBM, USA).

## Results

### Taxonomic composition

**Fig 2:**
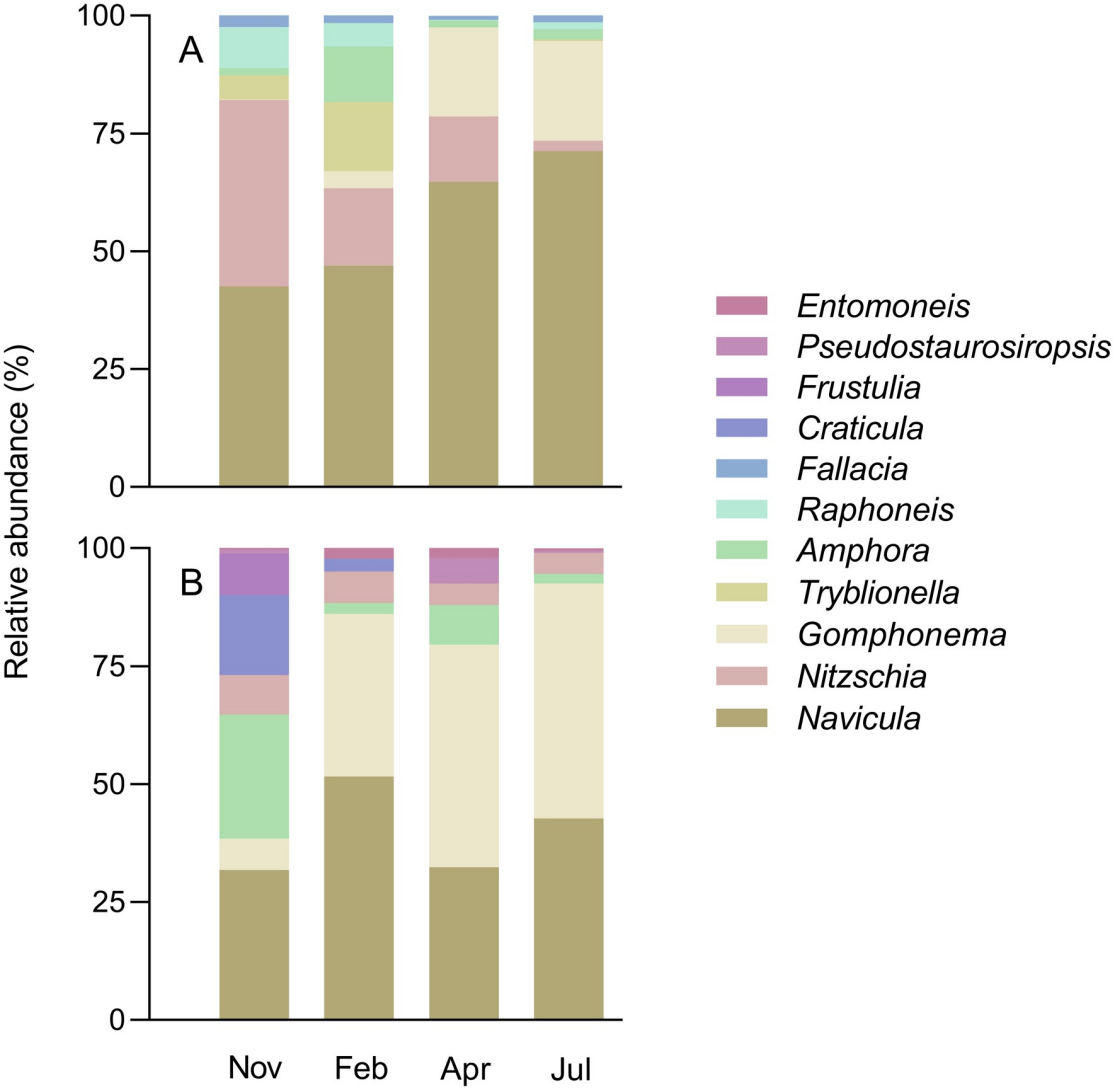
Taxonomical composition. Seasonal variation of the diatom composition of the VA-EPL (A) and GE-EPM (B) communities.

The microalgal communities in both sites were largely dominated by diatoms all year round (minimum 96.6%), but lower in VA-EPL (average 89.3%) than in GE-EPM (average 92%). The remaining taxa belonged to the euglenophytes (average 1.8%) and cyanobacteria (average 0.7%). Confirming previous studies, VA-EPL was dominated by motile forms, classifiable as EPL forms, while GE-EPM dominated by motile forms, classifiable as EPM. Nevertheless, several genera were found on the two sampling sites, the more abundant being *Navicula*, *Nitzchia*, *Gomphonema*, *Tryblionella* and *Amphora* (Fig 2). With the exception of VA-EPL in February and GE-EPM in November, two genera accounted for more than 50% of the cell counts, both for VA-EPL and GE-EPM. The muddy site VA-EPL was dominated all year round by diatoms of the genus *Navicula* (between 31.6% in November and 55.7% in July), which also dominated the communities of the sandy site in November and February (between 23.2% in April and 41.1% in February) (Fig 2). However, the communities of the two sites differed regarding the second most abundant genus. In in VA-EPL, this was *Nitzchia*, that showed highest abundance in November (29.4%) and decreased steadily towards summer (1.8% in July); in contrast, this genus had very low and relatively constant abundance in the GE-EPM communities (3.3% in July to 6.2% in November). In GE-EPM, the second most abundant genus was *Gomphonema*, with the lowest abundance in November (4.9%) and highest abundances in Abril and July (33.7% and 39.8%, respectively); in VA-EPL, this genus showed residual abundances in November and February (0.1% and 2.5%, respectively) but reached significant values in Abril and July (15.7% and 16.6%, respectively). Genera with average abundances between 1.0 and 10.0% included *Tryblionella* (3.6%), *Amphora* (3.1%), *Raphoneis* (2.6%) and *Fallacia* (1.2%) in VA-EPL, and *Amphora* (7.2%), *Craticula* (3.7%), *Frustulia* (1.6%) and *Pseudostaurosiropsis* (1.2%), in GE-EPM. A large number of genera appeared in smaller numbers, often in only one of the sampling occasions. These include the genera *Gyrosigma*, *Surirella* and *Cylindroteca* (VA-EPL) or *Diploneis*, *Fragillaria* and *Coconeis* (GE-EPM).

### Photoacclimation state

**Fig 3:**
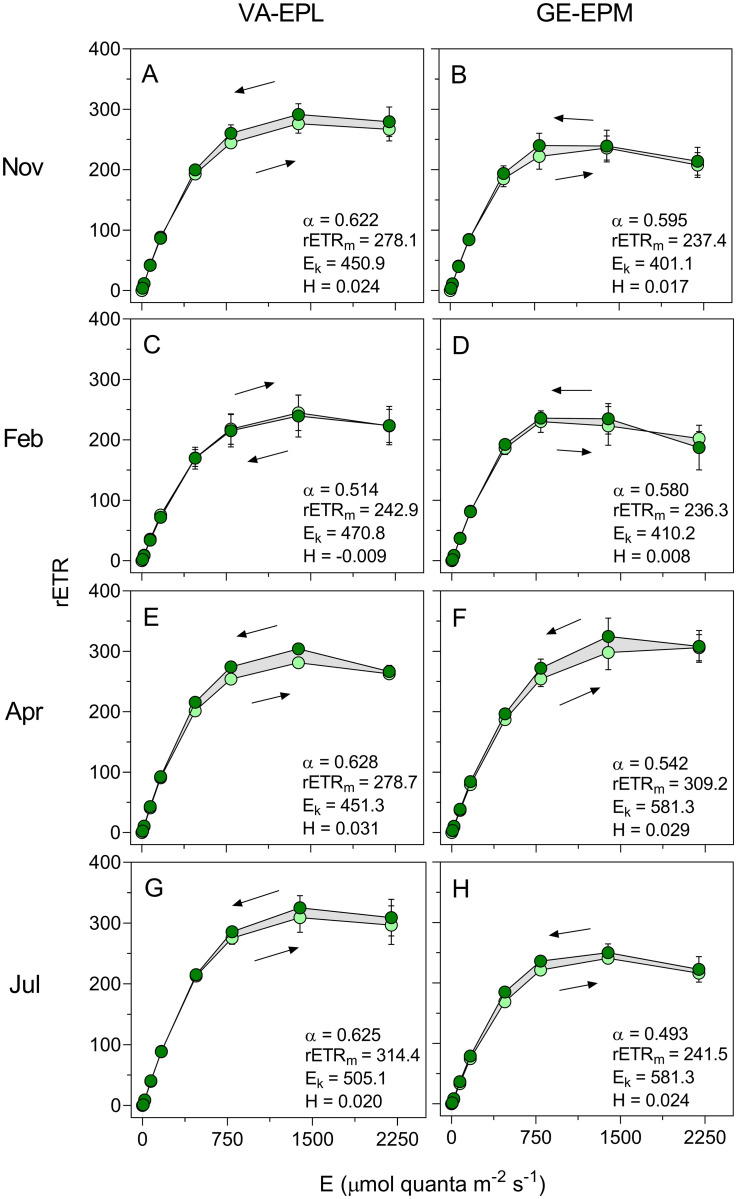
Seasonal variation of the photoacclimation state: rETR. Hysteresis light curves (HLC) of relative electron transport rate of PSII (rETR) of samples dominated by epipelic (VA-EPL) (A, C, E, G) and epipsammic (GE-EPM) (B, D, F, H) diatoms. Arrows indicate the order in which the measurements were carried out. Lighter and darker symbols represent measurements made during the light increase and the light decrease phases of the HLC, respectively. Mean values of three replicated HLCs. Error bars represent one standard error. Lines represent the model of Eilers and Peeters (1988) fitted to the mean values of rETR. The numbers indicate the parameters of the model fitted to the upward LC. The gray area indicates the difference between the integrated upward and downward LCs used to calculate the hysteresis index H.

As inferred form the upward phase of the HLCs of rETR, both types of communities appeared as high light-acclimated all year round (Fig 3). Photosynthetic activity, as denoted by rETR, saturated at high irradiance levels (E_k_ varying between 401.1 and 581.3 μmol quanta m^-2^ s^-1^) (Table 2), reached optimum values at around 1500 μmol quanta m^-2^ s^-1^, and showed only a slight decline for the highest irradiance level (E = 2250 μmol quanta m^-2^ s^-1^) (Fig 3). The light response of rETR was similar across sampling sites and seasons, and no significant differences were found between sites and seasons for both ETR_m_ and E_k_ (ANOVA, P > 0.089 in all cases), although there was a tendency for lower E_k_ values in Autumn and Winter and higher in Spring and Summer, at both sites. The VA-EPL samples appeared generally acclimated to higher light, as, with the exception of April, they showed higher rETR_m_ and E_k_ values. However, significant differences were found only for α, with VA-EPL samples showing higher mean values than the GE-EPM ones (ANOVA, F_1,24_, P = 0.018).

**Table 2:** rETR and Y(NPQ). Seasonal variation of the parameters of rETR and Y(NPQ) hysteresis light-response curves measured in EPL- and EPM-dominated communities. Parameter values are given for upward and downward light curves separately. Mean values of three replicated measurements ± one standard error.

			rETR				Y(NPQ)			
			α	rETR_m_	*E*_k_	*H*_rETR_	Y(NPQ)_m_	*E*_50_	n	*H*_Y(NPQ)_
VA-EPL	Nov	Up	0.622 ± 0.035	278.1 ± 16.3	450.9 ± 40.3	0.024 ± 0.007	1.210 ± 0.281	2632.3 ± 1440.5	0.86 ± 0.14	0.045 ± 0.008
		Down								
	Feb	Up	0.514 ± 0.018	236.8 ± 29.8	470.8 ± 51.2	-0.009 ± 0.002	2.132 ± 0.130	9704.8 ± 2107.8	0.45 ± 0.06	0.030 ± 0.004
		Down								
	Apr	Up	0.629 ± 0.035	282.0 ± 0.9	451.3 ± 24.9	0.031 ± 0.002	0.594 ± 0.010	974.2 ± 20.1	2.98 ± 0.09	0.027 ± 0.000
		Down								
	Jul	Up	0.625 ± 0.012	314.4 ± 25.1	505.1 ± 50.0	0.020 ± 0.006	0.562 ± 0.016	1096.1 ± 66.4	3.12 ± 0.12	0.038 ± 0.016
		Down								
	All	Up	0.597 ± 0.019	279.3 ± 11.8	469.5 ± 19.5	0.016 ± 0.006	1.124 ± 0.203	3601.9 ± 1210.0	1.85 ± 0.37	0.035 ± 0.004
		Down								
GE-EPM	Nov	Up	0.595 ± 0.031	237.4 ± 22.2	401.1 ± 42.4	0.017 ± 0.044	0.613 ± 0.008	841.1 ± 78.7	2.08 ± 0.15	0.071 ± 0.002
		Down								
	Feb	Up	0.580 ± 0.016	236.3 ± 23.5	410.2 ± 50.5	0.008 ± 0.007	0.650 ± 0.074	916.3 ± 51.3	2.49 ± 0.41	0.022 ± 0.003
		Down								
	Apr	Up	0.542 ± 0.037	309.2 ± 23.9	581.3 ± 80.9	0.029 ± 0.007	0.566 ± 0.023	1031.0 ± 92.2	2.71 ± 0.33	0.104 ± 0.025
		Down								
	Jul	Up	0.493 ± 0.030	241.5 ± 7.0	493.3 ± 31.9	0.024 ± 0.013	0.665 ± 0.022	1063.4 ± 57.4	1.87 ± 0.01	0.071 ± 0.004
		Down								
	All	Up	0.553 ± 0.017	256.1 ± 12.7	471.5 ± 32.0	0.019 ± 0.004	0.623 ± 0.021	963.0 ± 40.7	2.29 ± 0.15	0.067 ± 0.010
		Down								

Hysteresis was in all cases very low, the H index varying between -0.009 (VA-PL, February) and 0.031 (VA-EPL, April) (Fig 3, Table 2). Only in one case (VA-EPL, February) the hysteresis was negative, and only slightly (H = -0.009). No significant differences were found between the mean H values of the two sites (ANOVA, F_1,24_, P = 0.600), but a significant variation across seasons was detected (ANOVA, F_3,24_, P = 0.012). This seasonal variation was due to minimum H values in February, and comparable values during the rest of the year, in both sampling locations.

**Fig 4:**
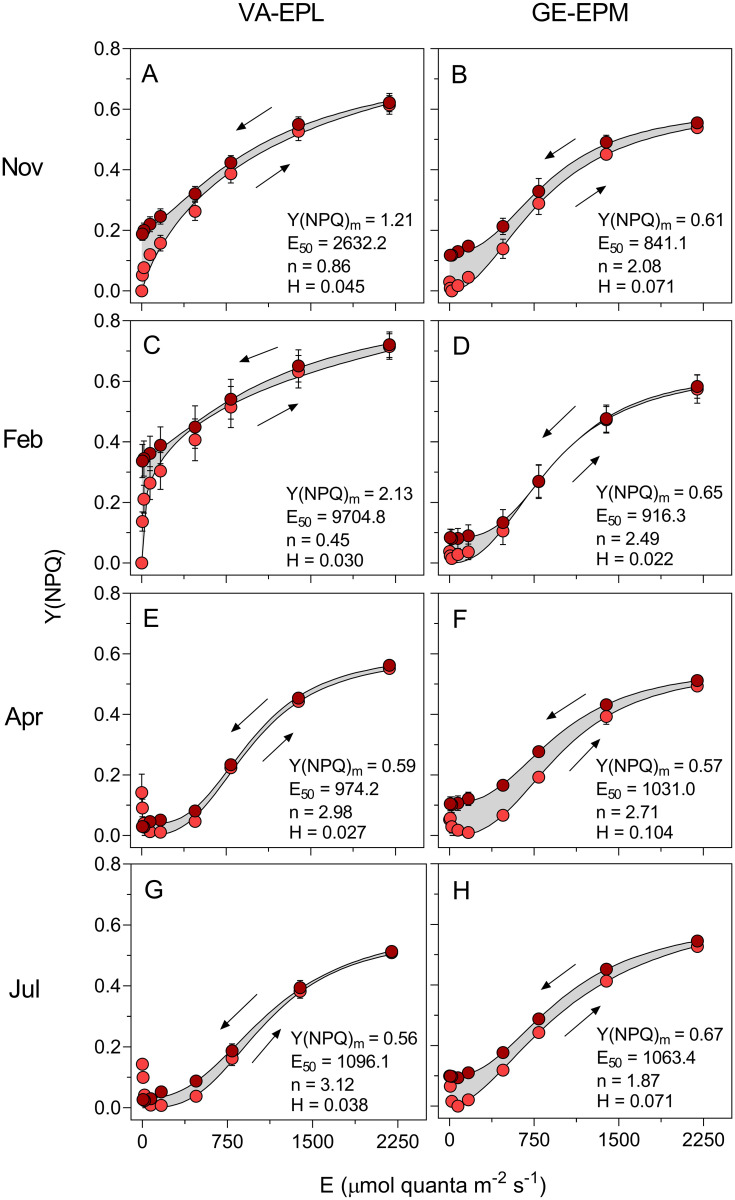
Seasonal variation of the photoacclimation state: Y(NPQ). Hysteresis light curves of the non-photochemical index Y(NPQ) of samples dominated by epipelic (VA-EPL) (A, C, E, G) and epipsammic (GE-EPM) (B, D, F, H). Arrows indicate the order in which the measurements were carried out. Lighter and darker symbols represent measurements made during the light increase and the light decrease phases of the HLC, respectively. Mean values of three replicated HLCs. Error bars represent one standard error. Lines represent the model of Serôdio and Lavaud (2011) fitted to the mean values of high light induced NPQ measurements. The numbers indicate the parameters of the model fitted to the upward LC. The gray area indicates the difference between the integrated upward and downward LCs used to calculate the hysteresis index H.

The HLCs of Y(NPQ) confirmed that the communities of the two sites were capable of dealing well with high light exposure, consistent with the high light photophenotype inferred from the rETR HLCs. Y(NPQ) HLCs did not saturate until the maximum irradiance applied, and, in various cases, the estimated values of Y(NPQ)_m_ were much higher than the highest measurement of the HLC, especially when these were highly sigmoid (e.g. VA-EPL, November and February) (Fig 4, Table 2). The E_50_ values were also high (in all but one case, above 900 μmol quanta m^-2^ s^-1^, GE-EPM), indicating the adjustment of the activation of NPQ processes to high light levels.

The VA-EPL samples showed a more pronounced high light-acclimated state, as denoted by the generally higher value of NPQ_m_ (exception: July) and E_50_ (exception: April). However, no significant differences were found between sites and seasons regarding E_50_ (ANOVA, *p*>0.065 in all cases). In contrast, significant variations were found for both Y(NPQ)_m_ and n between seasons (ANOVA, F_3,24_, *p* = 0.012 and ANOVA, F_3,24_, *p*<0.001, respectively) but not between sampling sites (ANOVA, F_11,24_, *p* = 0.061 and ANOVA, F_1,24_, *p*<0.210, respectively). For both VA-EPL and GE-EPM, Y(NPQ)_m_ tended to reach higher values in November and February, and lower values in April and July. Regarding the index n, a strong seasonal variation was observed mostly in VA-EPL samples, with higher sigmoidicity in April and July and lower in November and February while for GE-EPM samples a high sigmoidicity was present all year round. ‘Dark NPQ’ patterns were observed in spring and summer, especially marked in VA-EPL samples, associated to highly sigmoid curves (Fig 4E and 4G). This pattern was only observed in the light-increasing phase of the HLCs, being dissipated after exposure to high light (Fig 4E–4H).

HLCs of Y(NPQ) generally showed higher hysteresis levels than the rETR ones. Hysteresis was however still relatively low (and always positive), with H varying between 0.027 and 0.104, indicating a capacity for fast activation and relaxation of NPQ (Fig 4, Table 2). Significant differences were found between sites (ANOVA, F_1,24_, *p*<0.001) and between seasons (ANOVA, F_3,24_, *p* = 0.009), as well as an interaction between the two factors (ANOVA, F_3,24_, *p* = 0.007). Apart from February, the H index was always higher in GE-EPM than in VA-EPL samples.

### Seasonal variation of PSII photoinactivation and repair

**Fig 5:**
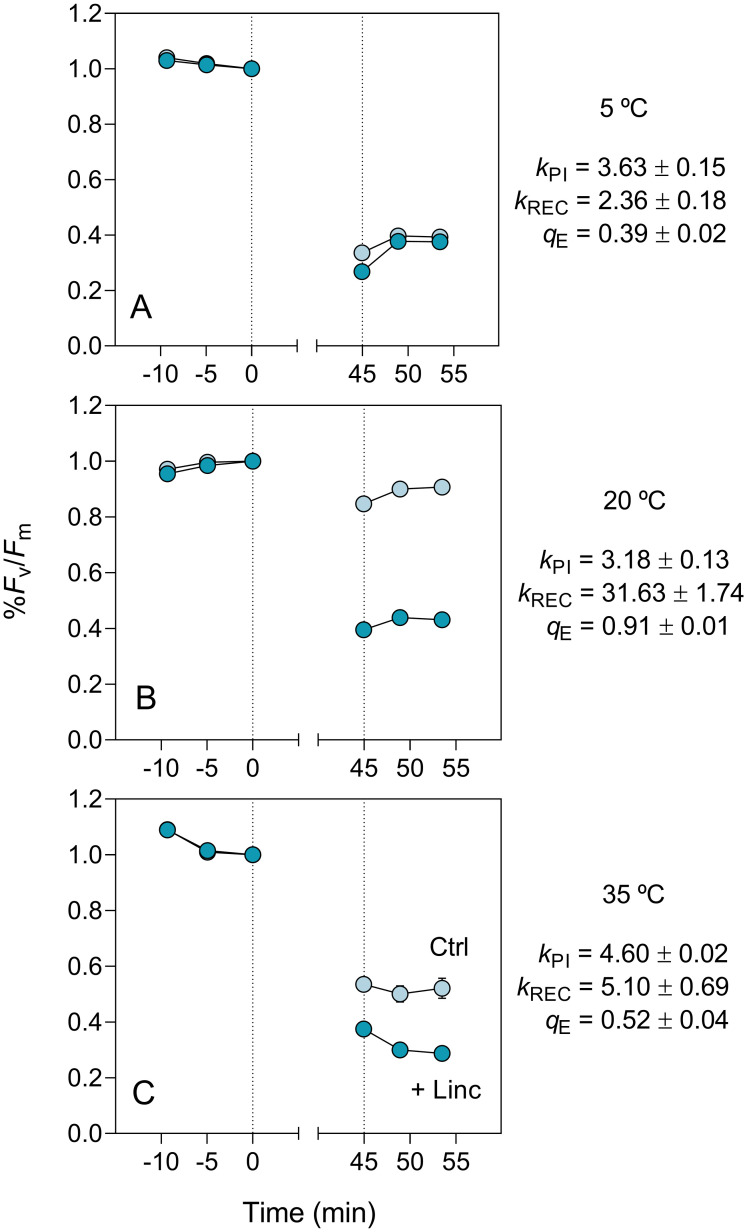
Light stress-recovery experiments. Illustrative results showing the impact of exposure to 1020 μmol quanta m^-2^ s^-1^ for 45 min (area between dotted lines) on %F_v_/F_m_ in control (untreated; Ctrl) and lincomycin-treated (+Linc) samples incubated at 5 (A), 20 (B) and 35 (C) °C. Data for GE-EPM in November. Units of k_PI_ and k_REC_: 10^−4^ s^-1^.

Fig 5 illustrates the results of LSE for the different tested temperatures (GE-EPM, November). The results for 20°C (Fig 5B) exemplify the main pattern that was observed in these experiments. Before the light stress, %F_v_/F_m_ stabilized as the samples acclimated to the test temperature and darkness, with no significant differences between lincomycin-treated and untreated samples. After the high light exposure, %F_v_/F_m_ showed a short-term relaxation reaching an apparent steady state within 15 min. A clear difference was observed between lincomycin-treated samples and controls. The former recovered to values of only 43% of pre-stress values, which resulted in k_PI_ = 3.2 × 10^−4^ s^-1^; the latter recovered almost completely, to values around 91% of pre-stress levels. This large difference is indicative of a large capacity for PSII repair, resulting in a high k_REC_ value (31.6 × 10^−4^ s^-1^), considerably higher than the corresponding k_PI_. The large recovery of the untreated samples is also indicative of an efficient photoprotection, quantified by q_E_ = 0.91.

This figure also exemplifies the effects of temperature on the rates of PSII photoinactivation and repair. The effects of temperature on k_PI_ were small, as seen by the comparable levels of %F_v_/F_m_ of lincomycin-treated samples, and the resulting values of k_PI_ = 3.6 and 4.6 × 10^−4^ s^-1^, for 5 and 35°C, respectively, not very different from the value measured at 20°C. In contrast, much larger and diverse effects were observed for k_REC_, with cold causing larger effects than moderate heat: under 5°C, there were almost no differences between untreated and lincomycin-treated samples, denoting a low repair capacity (k_REC_ = 2.36 × 10^−4^ s^-1^); for 35°C, a larger difference between controls and inhibited samples was observed which, although not as large as measured under 20°C, also indicated a lowered repair capacity (k_REC_ = 5.1 × 10^−4^ s^-1^). The smaller recovery observed for untreated samples under 5 and 35°C might also indicate a reduced photoprotection capacity, as compared to 20°C.

**Fig 6:**
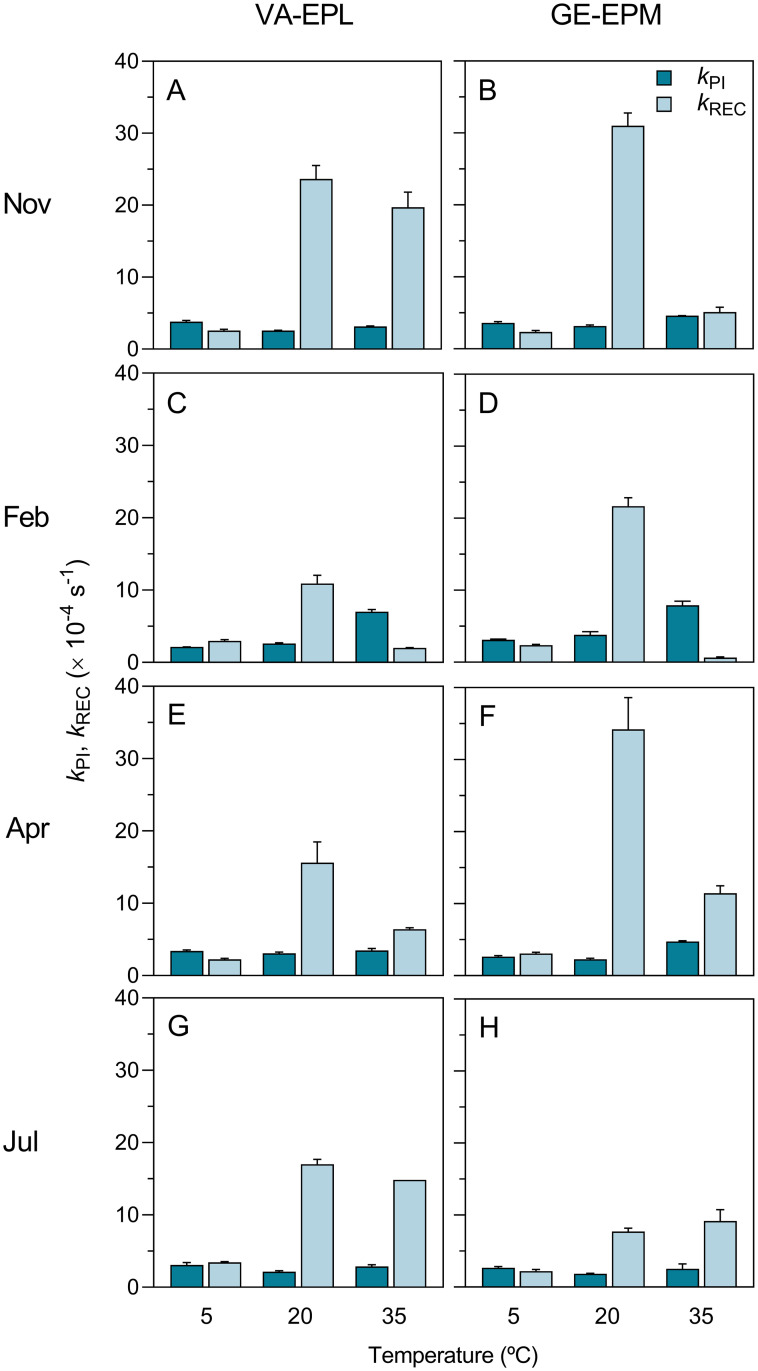
Seasonal variation of the effects of thermal stress: k_PI_ and k_REC_. Response to cold (5°C) and heat (35°C) stress of the rate constants of photoinactivation (k_PI_) and repair (k_REC_) of PSII of VA-EPL (A, C, E, G) and GE-EPM (B, D, F, H) communities.

Considering the entire dataset, the mean annual values of the k_PI_ observed for the stress-free conditions (20°C) on VA-EPL and GE-EPM samples were very similar (2.61 and 2.78 × 10^−4^ s^-1^, respectively; non-significant differences, ANOVA, F_1,24_, *p* = 0.199) and close to the overall average of 2.70 × 10^−4^ s^-1^ (Fig 6; Table 3). Significant differences were found between seasons (ANOVA, F_3,24_, *p*<0.001), with lowest k_PI_ values occurring in July (both sites) and highest values in February (GE-EPM) and April (V-EPL) (Table 3). GE-EPM samples showed a larger seasonal variation than VA-EPL ones, with mean k_PI_ values ranging from 1.86 (July) to 3.82 (February), a 105% variation; in contrast, VA-EPL showed a variation of 44% between minimum and maximum values (Fig 6; Table 3).

**Table 3:** k_PI_, k_REC_, Φ_PI_ and q_E_. Seasonal variation of the rate constant of PSII photoinactivation (k_PI_, × 10^−4^ s^-1^) and repair (k_REC_, × 10^−4^ s^-1^) and of the relative quantum yield of photoinactivation (Φ_PI_, × 10^−7^ m^2^ μmol quanta^-1^), measured in EPL- and EPM-dominated communities at 5, 20 and 35°C. Mean values of three replicated measurements ± one standard error.

Temperature		VA-EPL				GE-EPM			
(°C)		k_PI_	Φ_PI_	k_REC_	q_E_	k_PI_	Φ_PI_	k_REC_	q_E_
5	Nov	3.83 ± 0.16	3.75 ± 0.12	2.58 ± 0.16	0.40 ± 0.02	3.63 ± 0.15	3.56 ± 0.13	2.36 ± 0.18	0.39 ± 0.02
	Feb	2.14 ± 0.02	2.10 ± 0.02	2.99 ± 015	0.58 ± 0.01	3.11 ± 0.10	3.05 ± 0.10	2.37 ± 0.11	0.48 ± 0.01
	Apr	3.42 ± 0.13	3.35 ± 0.13	2.25 ± 0.13	0.40 ± 0.01	2.65 ± 0.14	2.60 ± 0.14	3.08 ± 0.18	0.54 ± 0.02
	Jul	3.07 ± 0.35	3.01 ± 0.35	3.44 ± 0.10	0.53 ± 0.01	2.67 ± 0.19	2.61 ± 0.19	2.20 ± 0.25	0.45 ± 0.03
	All	3.11 ± 0.21	3.05 ± 0.20	2.82 ± 0.15	0.48 ± 0.02	3.01 ± 0.14	2.96 ± 0.13	2.50 ± 0.13	0.46 ± 0.02
20	Nov	2.59 ± 0.04	2.54 ± 0.04	23.64 ± 1.86	0.90 ± 0.01	3.18 ± 0.13	3.12 ± 0.13	31.03 ± 1.74	0.91 ± 0.01
	Feb	2.60 ± 0.13	2.55 ± 0.13	10.93 ± 1.13	0.81 ± 0.02	3.82 ± 0.42	3.74 ± 0.41	21.69 ± 1.17	0.85 ± 0.01
	Apr	3.09 ± 0.15	3.03 ± 0.15	15.60 ± 2.89	0.97 ± 0.01	2.28 ± 0.13	2.23 ± 0.13	34.23 ± 4.40	0.94 ± 0.01
	Jul	2.15 ± 0.12	2.10 ± 0.11	17.03 ± 0.66	0.89 ± 0.00	1.86 ± 0.07	1.82 ± 0.07	7.72 ± 0.47	0.81 ± 0.01
	All	2.61 ± 0.11	2.56 ± 0.11	16.80 ± 1.59	0.89 ± 0.01	2.78 ± 0.25	2.73 ± 0.25	23.67 ± 3.28	0.87 ± 0.02
35	Nov	3.15 ± 0.06	3.09 ± 0.06	19.70 ± 2.10	0.86 ± 0.01	4.60 ± 0.02	4.51 ± 0.02	5.10 ± 0.69	0.52 ± 0.04
	Feb	7.03 ± 0.27	6.90 ± 0.17	2.01 ± 0.03	0.22 ± 0.00	7.90 ± 0.58	7.74 ± 0.56	0.64 ± 0.12	0.23 ± 0.02
	Apr	3.48 ± 0.27	3.41 ± 0.26	6.41 ± 0.22	1.14 ± 0.01	4.74 ± 0.12	4.64 ± 0.12	11.45 ± 1.03	0.71 ± 0.02
	Jul	2.88 ± 0.24	2.82 ± 0.23	14.85 ± 0.01	1.01 ± 0.01	2.56 ± 0.64	2.51 ± 0.63	9.18 ± 1.56	0.85 ± 0.08
	All	4.14 ± 0.52	4.05 ± 0.51	9.92 ± 2.28	0.81 ± 0.11	4.95 ± 0.61	4.85 ± 0.59	6.36 ± 1.33	0.58 ± 0.07

The k_REC_ values were in all cases much higher (varying from 4.2 to 15.0 times) than the corresponding k_PI_, reaching mean values of 16.80 and 23.67 × 10^−4^ s^-1^ for VA-EPL and GE-EPM, respectively. Significant differences were found between sites (ANOVA, F_1,24_, *p*<0.001) and seasons (ANOVA, F_3,24_, *p*<0.001), with minimum values being observed in winter (10.93 × 10^−4^ s^-1^, VA) and summer (7.72 × 10^−4^ s^-1^, GE) (Table 3). k_REC_ also showed a larger seasonal variation than k_PI_, varying by 116% and 344% for VA-EPL and GE-EPM, respectively.

### Effects of temperature on PSII photoinactivation and repair

The effects of the exposure to low and high temperatures were very marked and generally characterized by increases in PSII photoinactivation (higher k_PI_) and decreases in PSII repair capacity (lower k_REC_) (Fig 6; Table 3). Exposure to cold conditions resulted in similar k_PI_ values in VA-EPL and GE-EPM samples (averaging 3.11 and 3.01 × 10^−4^ s^-1^, respectively; Table 3), which were not significantly different from each other (ANOVA, F_2,12_, P = 0.697). Exposure to heat also caused similar effects on the k_PI_ measured in the two types of samples (averaging 4.14 and 4.54 × 10^−4^ s^-1^, respectively; Table 3), not significantly different (ANOVA, F_2,12_, *p* = 0.318). k_PI_ did not vary significantly between 5 and 20°C (ANOVA, Tukey HSD, *p* = 0.563), but varied significantly between these two temperatures and 35°C (ANOVA, Tukey HSD, *p*<0.001). Regarding k_REC_, also similar responses were measured for VA-EPL and GE-EPM samples, both for exposure to cold (mean values of 2.82 and 2.66 × 10^−4^ s^-1^, respectively; Table 3) and heat (mean values of 9.92 and 6.36 × 10^−4^ s^-1^, respectively; Table 3; non-significant differences in both cases, ANOVA, F_2,12_, *p* = 0.121 and *p* = 0.218, respectively). k_REC_ varied significantly between the three tested temperatures (ANOVA, Tukey HSD, P < 0.05 in all cases).

**Fig 7:**
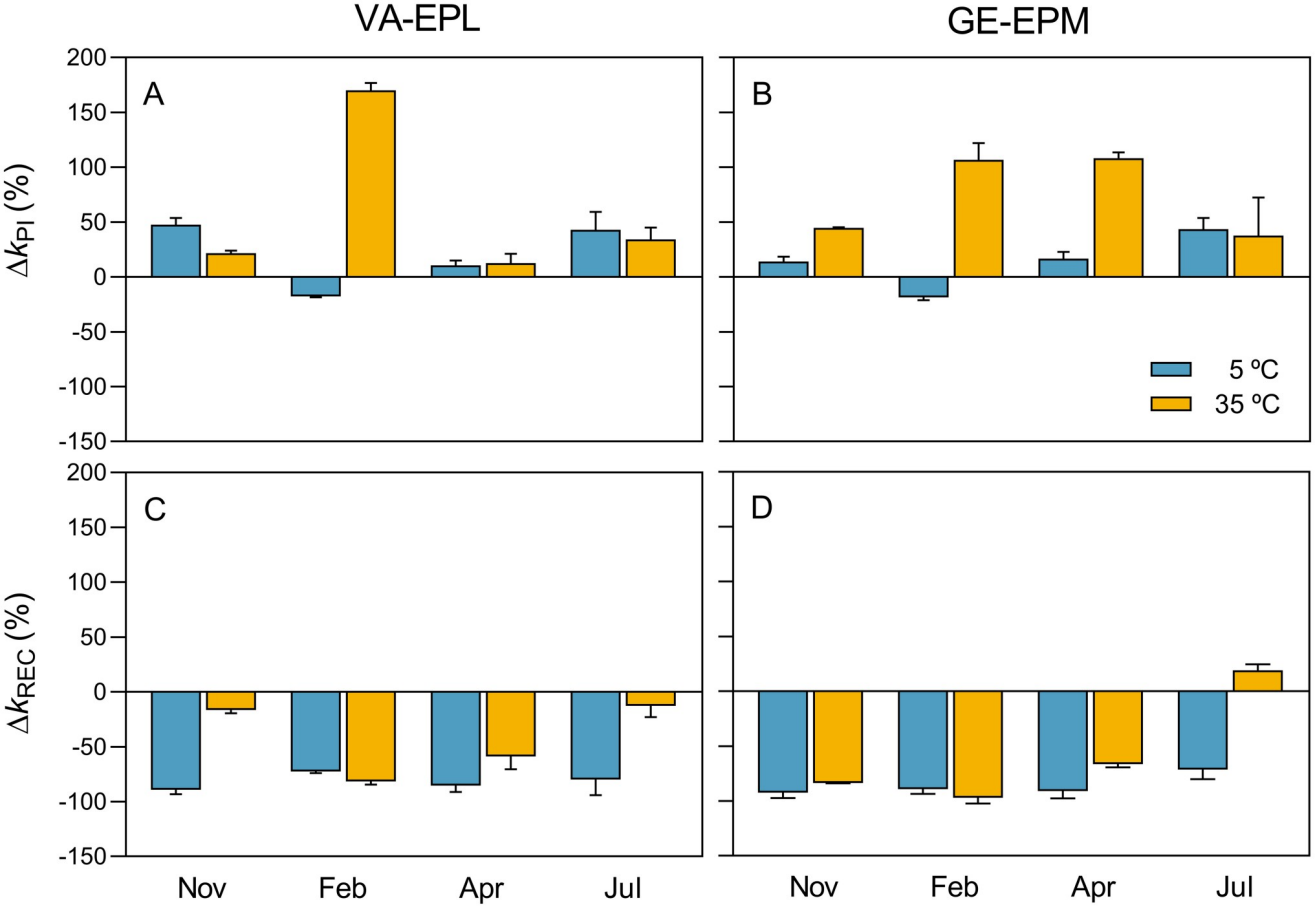
Seasonal variation of the effects of thermal stress: Δk_PI_ and Δk_REC_. Cold and heat-induced (5 and 35°C, respectively) change of the rate constants of photoinactivation (Δk_PI_) (A, B) and repair (Δk_REC_) (C, D) of PSII (in relation to values measured at 20°C) for VA-EPL (A, C) and GE-EPM (B, D) communities.

The effects of cold and moderate heat on k_PI_ and k_REC_ were highlighted by calculating the induced change relatively to 20°C (Δk_PI_ and Δk_REC_; Fig 7). This representation of the data reinforces the patterns described above, showing that cold caused overall larger effects on PSII repair capacity than on photoinactivation (average variation: +17% and -84% for k_PI_ and k_REC_, respectively) and heat caused comparable large effects on the two processes (average variation: +67% and -50% for k_PI_ and k_REC_, respectively).

Fig 7 also emphasizes that the effects of cold and heat exposure vary with the thermal acclimation state of the samples. The data show a tendency for stronger effects of heat exposure in winter, on cold-acclimated samples, and for stronger effects of cold exposure in summer, on heat-acclimated samples. Regarding k_PI_, exposure to cold generally caused an increase when compared to 20°C, reaching on average 20.78% and 13.9% for VA-EPL and GE-EPM, respectively. A well-defined seasonal pattern was observed, similar for VA-EPL and GE-EPM samples, characterized by higher k_PI_ values in July, on high temperature-acclimated samples, and lowest values in February, when samples were acclimated to cold conditions. (Fig 7A and 7B). Exposure to moderate heat caused in all cases an increase in k_PI_, with overall larger effects in GE-EPM (74.4%) than in VA-EPL samples (59.6%), and maximum values in winter and minimum values in autumn and summer. Most notable difference between the two sites regarded April, when k_PI_ was much lower in VA-EPL than in GE-EPM (Fig 7A and 7B). As pointed out above, overall effects on k_PI_ were larger under 35°C than under 5°C, reaching on average 67.0% and 17.3%, respectively.

Regarding k_REC_, cold exposure caused large decreases, similarly in VA-EPL and GE-EPM samples (-81.8% and -86.0%, respectively). Exposure to 35°C also caused similar effects on the two types of communities, reaching -57.0% and -42.5% for VA-EPL and GE-EPM, respectively (Fig 7C and 7D). A marked seasonal variation was evident for VA-EPL samples, with maximum effects in winter and spring, when samples were acclimated to low temperatures. For GE-EPM, minimum effects (the only positive change) were observed in July, when samples were acclimated to high temperatures (Fig 7C and 7D). Overall, exposure to cold caused stronger effects than exposure to heat, attaining on average -83.9% and -49.8, respectively. Cold- and heat-induced changes were globally higher for k_REC_ (-66.8%) than for k_PI_ (42.2%), and on average higher for GE-EPM (44.1% and -71.5%, for k_PI_ and k_REC_, respectively) than for VA-EPL (40.2% and -62.1%, for k_PI_ and k_REC_, respectively).

### PSII photoinactivation and repair vs photoprotection

Under non-stressed conditions (20°C), q_E_ reached high values (minimum value 0.81; Table 3), all year round in both sites, denoting an efficient capacity of the MPB communities to recover from high light stress. q_E_ was markedly affected by the temperature treatments, especially under cold conditions. Under 5°C, a generalized decrease was observed, with maximum value remaining below 0.6, and the lowest values recorded in November (Table 3). Under moderate heat, the response varied with season, with the lowest values being observed for February, in both sites (Table 3).

**Fig 8:**
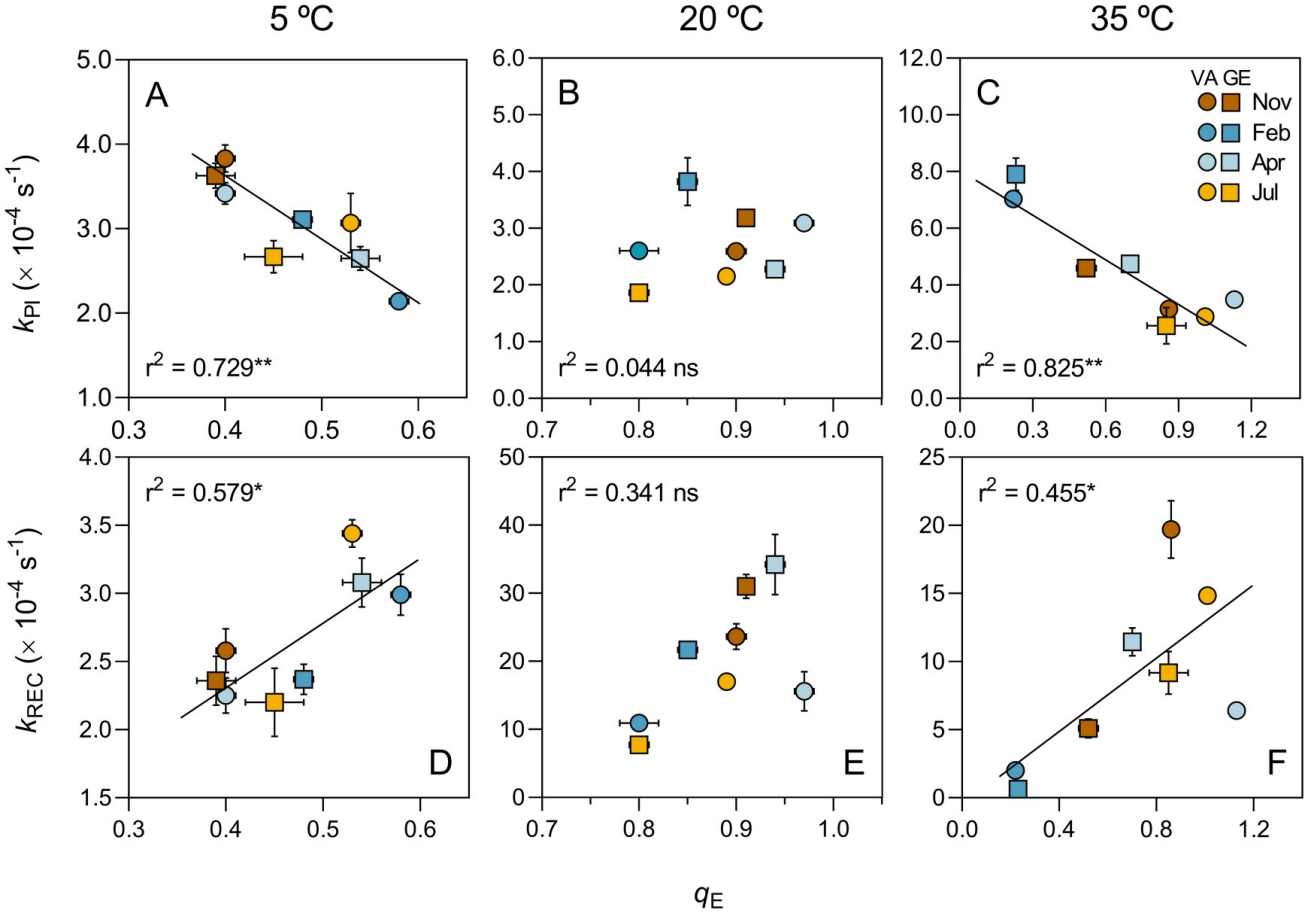
Rate constants vs NPQ. Relationship between the rate constants of photoinactivation (k_PI_; A-C) and repair (k_REC_; D-F) of PSII and energy-dependent quenching (q_E_) for samples exposed to 5 (A, D), 20 (B, E) and 35 (C, F) °C. Sampling site and occasion are identified by shape and color of data points, respectively.

Significant linear negative relationships were found between k_PI_ and q_E_ for both 5°C and 35°C, but not for 20°C (Fig 8A–8C), suggesting an important role of photoprotective mechanisms in preventing PSII photoinactivation under extreme temperatures. Significant linear relationships were also found between k_REC_ and q_E_ for both 5°C and 35°C, but not for 20°C (Fig 8D–8F). In this case, the relationships were positive, indicating a direct dependency of PSII repair on the photoprotection capacity under extreme temperature conditions. The linear relationships with q_E_ were stronger for k_PI_ than for k_REC_ (Fig 8A, 8C, 8D and 8F). No significant linear relationships were found between k_PI_ and k_REC_, for any of the tested temperatures.

## Discussion

### Variation of k_PI_ and k_REC_ with photoacclimation

One main objective of this work was to study how the susceptibility to PSII photoinactivation and the capacity for PSII repair, and their responses to extreme temperature, varies with the photoacclimation state of the MPB communities. The results showed that no substantial changes in the photoacclimation state occurred over the studied seasonal cycle. Both EPL and EPM communities appeared high light acclimated all year round, although the VA-EPL samples appeared generally more high light-acclimated than the GE-EPM ones. The high light-acclimated photophenotype was confirmed by the ability to cope well with the exposure to high light, supported by the hysteresis light-response curves of both rETR and Y(NPQ): in all cases HLCs showed a positive but low hysteresis, indicative of absence or small cumulative negative effects on photosynthesis [53], especially considering that LCs included E levels as high as 2250 μmol quanta m^-2^ s^-1^. As observed for rETR, the EPL communities generally displayed a more pronounced high light-acclimated state in terms of their light response of Y(NPQ).

The high-light acclimation state of the MPB of the Ria de Aveiro throughout the year has been reported before [2, 31]. Also the absence of substantial seasonal changes has been documented [59], although contradicted by posterior studies [2]. On the basis of this apparent contradiction is likely the large variability in the photosynthetic light response of natural samples, susceptible to large variability at sub-seasonal time scales (spring-neap cycle tidal; [2]). The differences between the mentioned studies can also be related to the methodology used to generate the LCs: seasonal differences were found when applying rapid LCs protocols [2] but not when measuring steady state LCs [59], as in the present study.

The more pronounced light-acclimation photophenotype of the VA-EPL communities, and the absence of clear seasonal patterns, can be explained by the behavioral regulation of light exposure, through vertical migration of the diatom cells within the thin photic zone of the sediment [20, 24]. By enabling cells to remain under light levels that maximize photosynthesis, their photobehavior may contribute to the maintenance of a marked high light-acclimation state all year round, independently of the seasonal change of incident solar light [35]. The large variability in the photoacclimation state on natural communities may also be the cause of the conflicting results that have been reported for the MPB of the VA-EPL and GE-EPM sites of the Ria de Aveiro. In the present work, in comparison with GE-EPM, the EPL-VA samples showed significantly higher values of α and of Y(NPQ)_m_, in agreement with one study [31] but not with another [2].

Despite the absence of large seasonal variations in photoacclimation state, both k_PI_ and k_REC_ varied significantly with the time of the year, indicating that the processes underlying changes in the susceptibility to PSII photoinactivation and in the PSII repair capacity may vary independently from the photosynthetic use of light. Nevertheless, the consistent observation of minimum k_PI_ values in July may indicate a beneficial effect of the exposure to high solar irradiance on reducing PSII photoinactivation.

The extent of photoinactivation suffered by the MPB communities measured in this study are aligned with data from previous studies. Converting the values of k_PI_ to the relative quantum yield of photoinactivation, Φ_PI_, a parameter independent from the E level applied that facilitates inter-studies comparisons (Φ_PI_ = k_PI_/E; [16]), the results reached in this study, 2.10 and 1.82 × 10^−7^ m^2^ μmol quanta^−1^ for VA-EPL and GE-EPM, respectively (July; Table 3), match closely, both in terms of relative (higher values for VA-EPL) and absolute values, previous estimates of 3.25 vs 1.27 × 10^−7^ m^2^ μmol quanta^−1^ for the same sites and season (August; [31]). The global range of Φ_PI_ determined in this study, varying between 2.10 and 3.74 × 10^−7^ m^2^ μmol quanta^−1^ (Table 3), also agree with data recently compiled for diatoms, averaging 3.72 × 10^−7^ m^2^ μmol quanta^−1^ [60].

Consistent across sampling sites and occasions was the finding that, under non-stress conditions (20°C), k_REC_ typically reached values much higher than the corresponding k_PI_, on average almost 10x higher (20.4 vs 2.70 × 10^−4^ s^−1^, respectively). This result may be interpreted as indicative of efficient repair mechanisms, allowing the cells to maintain a stable pool of active PSII [13]. Large PSII repair rates would explain the high recovery capacity demonstrated by the high q_E_ values generally measured across sites and seasons. Such high PSII repair capacity is advantageous when exposure to photoinhibitory conditions cannot be avoided, especially frequent in the intertidal environment. However, high k_REC_ values may result from the erroneous assumption, based on the Kok model, that there are no significant amounts of inactive PSII before the exposure to light stress (Campbell & Serôdio 2021). This may be the case in the present study, as there is evidence that diatoms are able to maintain a pool of inactive PSII as substrate for repair (Lavaud et al. 2016) [21].

### Effects of thermal stress on PSII photoinactivation and repair

The main novelty of this study was the attempt to quantify the effects of acute, short-term exposure to extreme temperatures on PSII photoinactivation and repair in natural MPB communities, simulating the sudden changes in temperature that often occur in the intertidal environment. The results revealed clear patterns of response to cold and heat stress, similar in both VA-EPL and GE-EPM communities, characterized by: (i) extreme temperatures affected both PSII repair capacity (decreasing k_REC_) and PSII photoinactivation (increasing in k_PI_); (ii) overall, the effects were higher on PSII repair capacity (large decreases in k_REC_) than on PSII photoinactivation (moderate increases in k_PI_); (iii) cold stress caused larger effects on the repair capacity (decrease of k_REC_) than on photoinactivation of PSII (increase of k_PI_); (iv) heat stress caused comparable large effects on the two processes.

These results are consistent with previous studies, carried out on a variety of organisms. Cold stress is known to increase PSII inactivation (increase in k_PI_) by enhancing various photoinhibitory mechanisms, such as the double reduction of the primary electron acceptor of PSII (Q_A_; acceptor-side mechanism) and single oxygen production from recombination processes [14]. Cold can also limit PSII repair (decrease in k_REC_) [13, 51], as it inhibits protein synthesis, including the target of photoinhibition, the PSII D1 protein [61]. Low temperatures affect especially the processing of pre-D1 protein, a step in the formation of mature D1 protein and in the assembly of the active PSII complex [62]. On the other hand, moderate heat stress aggravates net photoinhibition both through the direct inactivation of the oxygen-evolving complex (increase in k_PI_) and the inhibition of PSII repair (decrease in k_REC_) [62].

Despite these common overall patterns of variation of the two studied communities, GE-EPM samples tended to suffer larger impacts of cold and heat stress and to show a more pronounced seasonal variation than VA-EPL. Under non-stress conditions, the differences observed between the two communities are in agreement with previous results for the same sampling sites and time of the year (summer; [31]), with both k_PI_ and k_REC_ reaching higher values for VA-EPL, supporting the trade-off between motility and physiology. This pattern, however, was not observed for other seasons, when k_PI_ was higher in VA-EPL samples only in April, and k_REC_ was in all cases lower in VA-EPL than in GE-EPM samples. These results indicate that, with the exception of the summer months, the motility of EPL species is not associated to a lower physiological capacity for preventing photoinactivation but is associated to a lower capacity for PSII in comparison with non-motile EPM forms. Under cold and heat stress, the results generally agree with the abovementioned study regarding k_REC_ (higher values for VA-EPL, excepting cold stress in spring), but not regarding k_PI_: identical (cold stress) or higher (heat stress) values were reached in GE-EPM in comparison with VA-EPL.

The results on the relative importance of PSII photoinactivation and repair in motile and non-motile diatom forms under thermal stress force to revise and extend the trade-off hypothesis. A trade-off between motility- and physiology-based photoprotection appear to exist, not so much regarding the susceptibility to PSII photoinactivation (capacity for motility allows a lower physiological photoprotection) but mainly regarding PSII repair: motile forms have a higher inherent capacity for repair than the immotile forms. This aspect, reported before for room temperature [31], is shown in this study to be especially important under thermal stress. This seems ecophysiologically relevant, as in the intertidal environment extreme temperatures typically occur during low tide, when the cells are often also exposed to potentially photoinhibitory high light levels. The higher repair capacity of EPL forms may be associated to the use of light-driven motility to seek and remain under the high light conditions in the upper layers of the sediment, maximizing photosynthesis and growth [35]. The permanence in these layers for long periods requires the capacity to cope adequately with high light conditions, consistent with the particularly accentuated high light-acclimation state observed of EPL samples, for which an efficient PSII repair capacity is clearly advantageous.

While seasonal changes in PSII photoinactivation and repair rates were not strongly associated to changes in photoacclimation state, the results suggest that changes in thermal acclimation throughout the year may have mediated the responses to cold and heat treatments. In fact, heat exposure tended to cause stronger effects in winter, when cells are acclimated to low ambient temperatures, while the cold stress tended to cause stronger effects in summer, when cells were expected to be acclimated to high ambient temperatures. Thermal acclimation has been shown to have effects on both PSII susceptibility to photoinactivation [63] and on PSII repair rates [51], although the underlying mechanisms are not yet identified.

### PSII photoinactivation and repair vs photoprotection capacity

The absence of an association between q_E_ and both k_PI_ and k_REC_ under thermal stress-free conditions is an indication that changes in PSII photoinactivation and repair occur independently of the photoprotection capacity of both EPL and EPM communities. This means that photoprotection conferred by NPQ processes, presumedly very efficient considering the high values of q_E_, is not sufficient to effectively protect the cells from increases in PSII photoinactivation or decreases in PSII repair caused by high light exposure. This non-correlated variation between q_E_ and k_PI_ and k_REC_ may be also due to the relatively small range of variation of qE amongst sites and seasons that was observed under 20°C.

In contrast, the significant correlations found between q_E_ and both k_PI_ and k_REC_ under cold and heat conditions suggest a strong effect of changes in photoprotection capacity on PSII photoinactivation and repair processes. This is supported by the observation that the decrease in photoprotection capacity, caused by the acute stress induced by cold and heat exposure, is followed by a substantial increase in susceptibility to photoinactivation and decrease in repair capacity. The inverse relationships observed between k_PI_ and q_E_ for both 5 and 35°C support that a higher photoprotection capacity contributes to reducing the PSII photoinactivation caused by thermal stress. On the other hand, the positive relationships found between k_REC_ and q_E_ suggest a direct dependency of PSII repair on photoprotective mechanisms. These appear to protect the PSII repair process from the effects of thermal stress, as the decrease in repair capacity under cold and heat exposure is alleviated under high q_E_. These results indicate that photoprotection processes have an important role under acute thermal stress when the limitation of PSII photoinactivation and the activation of repair processes is more needed.

### Mode of action of abiotic stress

Photoinhibition induced by abiotic stress has been traditionally viewed as resulting from the direct action of ROS in promoting PSII inactivation. However, recent experimental evidence has suggested a ‘new paradigm’ of photoinhibition, according to which: (i) abiotic stressors, including cold and moderate heat, act primarily by inhibiting or decelerating the repair of damaged PSII rather than by causing significant direct PSII photoinactivation; (ii) photoprotective mechanisms act mainly by protecting PSII repair from ROS action and not by preempting PSII photoinactivation [62, 64, 65]. The results of this study shed light on the validity of this hypothesis on benthic diatoms. Although effects differ between cold and moderate heat exposure, in both cases acute thermal stress was found to cause photoinhibition and impact photosynthetic activity both through increase in susceptibility to photoinactivation and inhibiting repair. The new scheme is not supported by the observation of significant direct effects on k_PI_ and by the comparable large effects on k_PI_ and k_REC_ (with tendency for stronger impacts on the former) caused by heat stress. On the other hand, cold stress affects more severely PSII repair capacity than photoinactivation, a result aligned with the new paradigm. Also contrary to new scheme is the observation that photoprotection effectively reduces PSII photoinactivation, although a positive effect on repair was also observed. It may thus be concluded that, regarding MPB diatoms, the ‘old’ and ‘new’ schemes are not mutually exclusive, but that an intermediate state between these two extremes is the one that better describes the responses of these natural communities to thermal stress.

## Supporting information

S1 File STL file for 3D-printing the custom-designed cuvette holder and water jacket. (STL)
